# Pegbovigrastim use in periparturient embryo-recipient cows: Effects on health and reproduction

**DOI:** 10.3168/jdsc.2022-0285

**Published:** 2023-02-09

**Authors:** D. Cortes, M. Chirivi, S. Wang, G.A. Contreras

**Affiliations:** 1 Department of Large Animal Clinical Sciences, Michigan State University, East Lansing 48824; 2 Center for Statistical Training and Consulting, Michigan State University, East Lansing 48824

## Abstract

The objective of this randomized clinical study was to evaluate the effects of periparturient administration of pegbovigrastim (recombinant bovine granulocyte colony stimulating factor; PEG) on postpartum health and reproductive outcomes in dairy cows carrying transferred embryos. A total of 60 multiparous Jersey cows (second parity n = 21, >second parity n = 39) that were in vitro-fertilized embryo recipients were enrolled. Animals were selected from a commercial herd and assigned to 1 of 2 treatments: (1) PEG (n = 31), which received a subcutaneous injection of 15 mg of PEG 7 d before expected calving and a second dose within 1 h of parturition; or (2) CON, untreated control (n = 29). Parturition was induced pharmacologically in all cows at 280 d of pregnancy with 500 μg of cloprostenol and 25 mg of dexamethasone intramuscularly. Cow health was monitored daily during the periparturient period and included assessment for retained fetal membranes within 24 h after calving and metritis until 20 d in milk. Descriptive statistics were calculated, to determine the incidence of postpartum diseases, causes of culling, and early-lactation reproductive outcomes, including number of inseminations, days to first heat, days to first service, and interval from calving to conception. The Wilcoxon rank sum test was used to assess treatment effects on early-lactation reproductive performance outcomes. The overall incidence of metritis in this cohort of cows was 17.14%. Animals treated with PEG tended to have more cases of metritis (25%) compared with CON (8.8%). Mastitis incidence in PEG was 19.35% compared with CON at 27.59%. These data suggest that in this dairy, with embryo-recipient cows induced to calve at 280 d of pregnancy, the administration of 2 doses of PEG tended to increase postpartum metritis but had no effect on mastitis incidence, culling rate, or early-lactation reproductive outcomes.

## Highlights

• Pegbovigrastim (PEG) tends to increase metritis incidence in embryo-recipient Jersey cows induced to calve pharmacologically.
• PEG does not affect the incidence of metabolic diseases, culling rate, or reproductive outcomes in multiparous Jersey cows induced to calve at 280 d of pregnancy with 500 μg of cloprostenol and 25 mg of dexamethasone intramuscularly.
• Findings reported in this study are from a population of mature, embryo-recipient Jersey cows, which are often not represented in larger clinical trials and observational studies evaluating the effects of PEG.

## Graphical Abstract

**Figure:**
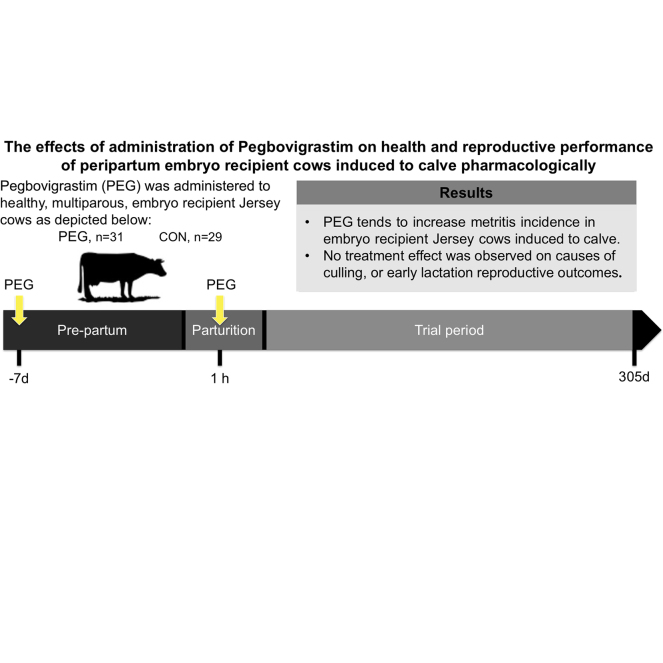
**Summary:** Metritis is a common disease in dairy cows during the postpartum period and is associated with immune system impairment. Embryo-recipient cows are reported to have delayed parturition and are at a higher risk for retained fetal membranes and metritis. This study evaluated the effects of pegbovigrastim (PEG), an immunomodulator, on health and reproductive outcomes in 60 embryo-recipient Jersey cows induced to calve at 280 d of pregnancy. Animals were assigned to PEG-treated or untreated control groups. Cows treated with PEG tended to have a higher incidence of metritis by 20 DIM. No treatment effect was observed on postpartum disease incidence, culling rates, or reproductive outcomes.

Metritis is a common postpartum disease in cattle characterized by a malodorous, sanguinopurulent uterine discharge and an enlarged uterus. In the United States, this disease affects 18.5 to 20% of lactating cows during the first 3 wk after calving (Sheldon et al., 2008; Galvão, 2012). Often, the uterine symptoms are accompanied by delayed uterine involution and signs of systemic illness such as depression, anorexia, fever (>39°C), and decreased milk production (Sheldon et al., 2006; Galvão, 2012). Furthermore, metritis has a negative impact on farm profitability due to production losses, poor reproductive performance, and increased culling rate. The calculated average mean cost per case of metritis is $513 USD (Pérez-Báez et al., 2021). Risk factors for metritis include retained fetal membranes (**RFM**) and intense, protracted negative energy balance, which are often associated with immune dysfunction (Hammon et al., 2006; LeBlanc et al., 2011; Giuliodori et al., 2013).

In vitro-fertilized (**IVF**) embryo-recipient dairy cows have been reported to have delayed parturition and heightened risk for RFM and metritis (Beagley et al., 2010). In addition, IVF calves are of larger size and weight than those fertilized naturally, making their dams more susceptible to dystocia (Young et al., 1998). To address this issue, producers often induce parturition pharmacologically to ensure the live birth of embryo-transfer calves (Nasser et al., 2008). However, this intervention affects placental maturation and fetal cortisol surge, 2 processes that are essential for fetal membranes expulsion (Benedictus et al., 2011, 2015).

Placental expulsion is a complex process highly dependent on regulated inflammatory responses. One of the mechanisms of placental detachment is the breakdown of the extracellular matrix by maternal neutrophils, which release matrix metalloproteinase under the action of proinflammatory cytokines, such as IL-8 (Kimura et al., 2002; Nelli et al., 2019). Dairy cows are at a higher risk for RFM when periparturient immune cell dysfunction occurs and placental extracellular matrix breakdown is impaired (Galvão et al., 2010; Barca et al., 2022). For this reason, immunomodulators that potentiate the inflammatory response of immune cells, especially neutrophils and other polymorphonuclear cells, could increase the efficacy of immune function around parturition (Stabel et al., 1991).

Pegbovigrastim (**PEG**) is a recombinant bovine granulocyte colony stimulating factor bound to polyethylene glycol, which stimulates the differentiation of neutrophilic granulocytes in the bone marrow, leading to a sharp increase in blood neutrophil counts, with enhanced cytotoxic activity and myeloperoxidase secretion (Kimura et al., 2014; Freick et al., 2018). The activity of PEG may enhance the uterine inflammatory process, leading to fetal membranes expulsion; however, it is unknown whether excessive inflammation may increase the incidence of metritis in periparturient cows. Ruiz et al. (2017) reported that administration of PEG 7 d before expected calving and within 24 h after parturition increased metritis incidence by 17.6% during the first 21 DIM; this effect was due to an exacerbated neutrophil response. In their study, the timing of the second (postpartum) PEG administration, although it occurred during the first day, was highly variable hourly. Because the timing for the second PEG dose (postpartum) may be important due to its possible effects on the placental expulsion process, applying the dose closer to calving may improve the immunomodulatory effects of PEG on placental expulsion and uterine involution. We hypothesized that the administration of PEG at 7 d before expected calving and within 1 h postpartum will reduce the incidence of metritis in parturition-induced embryo-recipient dairy cows and improve their health and reproductive performance.

All animal procedures were approved by Institutional Animal Care and Use Committee of Michigan State University. A total of 60 healthy, multiparous, IVF embryo-recipient Jersey cows (second parity n = 21, >second parity n = 39) were selected from a Michigan commercial dairy farm. Cow enrollment took place from February to April of 2019. Cow inclusion criteria were: >250 d carrying a calf from IVF transplantation and no history of RFM or metritis in the previous lactation. At the time of enrollment, cows were randomly assigned to 1 of 2 treatments. The PEG group (n = 31) received 2 doses of PEG (15 mg administered s.c.) 7 d before expected calving and within 1 h after parturition. The CON group (n = 29) did not receive any medication. Parturition was induced in all cows at 280 d of pregnancy with 500 μg of cloprostenol and 25 mg of dexamethasone i.m. After induction, cows were monitored by farm personnel for 24 h for visual signs of labor. Following parturition, cows were moved into the fresh cow pen and kept until 20 DIM. Cows were monitored twice a day for periparturient diseases until 30 DIM by a trained herdsman. In this dairy, the case definitions for diseases are as follows. (1) Retained fetal membranes: fetal membranes not expelled within 24 h postpartum. (2) Metritis: presence of a watery fetid reddish uterine discharge confirmed by rectal palpation (0–20 DIM). (3) Clinical mastitis: presence of abnormal milk with swollen udder or systemic symptoms, or both (0–30 DIM). (4) Lameness: abnormal gait with pain or discomfort using the lameness scoring system of Sprecher et al. (1997), as follows: 1 = normal, 2 = mildly lame, 3 = moderately lame, 4 = lame, and 5 = severely lame (0–30 DIM). (4) Clinical ketosis: clinical symptoms such as depression, inappetence, lethargy, weight loss, decreased milk production, and positive ketone body urine strip test (Ketostix, Bayer Corp.). Any cow with trace urine ketones 15 mg/dL or higher was considered positive during the first 30 DIM. (5) Left displaced abomasum: inappetence, decreased milk production, and presence of a tympanic resonance sound “ping” during abdominal percussion (0–30 DIM). (6) Pneumonia: respiratory distress with nasal secretion or fever and depression (0–30 DIM). (7) Clinical hypocalcemia or milk fever: recumbency, inappetence, decreased milk production, and lethargy during the first 30 DIM. (8) Anestrus: no deviation of dominant follicle, regression of dominant follicle, or persistency of dominant follicle “follicular cyst” without ovulation, as assessed by transrectal ultrasonography using ultrasonography equipment with a rectal linear transductor of 5 to 7 MHz (Easy-Scan, IMV) by trained farm personnel during 60 to 120 DIM.

Health and reproductive events were recorded in the herd management software DairyComp305 (Valley Agricultural Software). Descriptive statistics were performed to determine the incidence of postpartum diseases, causes of culling, and early-lactation reproductive performance outcomes including number of inseminations, days to first heat, days to first service, and interval from calving to conception. Due to the skewed distributions of reproductive performance variables, Wilcoxon rank sum tests were used to compare CON and PEG groups. Statistical assessment was performed using R software (version 4.1.1, R Foundation for Statistical Computing).

Retained fetal membranes was not diagnosed in the first 24 h after parturition in the study population induced to calve at 280 d of pregnancy. This may be attributed to the advanced stage of pregnancy at which calving was induced. Previous studies reported a low incidence of RFM after calving was induced at advanced stages of pregnancy (>275 d), due to improved immune-assisted detachment in mature placenta (Benedictus et al., 2011). Our results contrast with those reported by Shenavai and colleagues (2012), who reported an association between induction of parturition before 275 d of gestation and high incidence of RFM. Differences between our study and that of Shenavai et al. (2012) may be related to differences in physiological placenta maturation due to a premature induction of the parturition in cows (Echternkamp and Gregory, 1999). The overall incidence of metritis during the first 20 DIM in this study was 17.14%. Similar findings were reported in previous studies, where the overall incidence of metritis in dairy cows was 21.7% in 83 multiparous Holstein cows in a single commercial farm (Hammon et al., 2006) and 18.6% across 5 commercial Israeli dairy herds (Goshen and Shpigel, 2006). The incidence of metritis was 25% in PEG compared with 8.8% in the CON group (*P* = 0.06). These results coincide with those reported by Ruiz et al. (2017) and Oliveira et al. (2020), where PEG-treated cows had a greater incidence of metritis compared with controls (17 and 25%, respectively). The difference in the incidence of metritis between groups could be related to the mechanism of action of PEG that induces a sharp increase in circulating neutrophil counts and their inflammatory activity (McDougall et al., 2017).

We found no differences in lameness incidence between PEG (13%) and CON (7%; Table 1). It is important to note that Zinicola et al. (2018) reported that Holstein dairy cows treated with PEG showed a higher incidence of lameness during the first 30 DIM, with 15.69% versus 9.68% in control cows. The mechanisms leading to higher lameness susceptibility in PEG-treated cows appear to be multifactorial and include bone marrow expansion, recruitment and stimulation of inflammatory cells, and bone resorption by osteoclast activity (Zinicola et al., 2018).

**Table 1:** Incidence of periparturient diseases in in vitro-fertilized pregnant cows induced to calve at 280 d of pregnancy assessed during the first 120 DIM^1^

Disease	Group (n = 60)			Proportion^2^		
	CON (n = 29)	PEG (n = 31)	DIM	CON	PEG	Dif
Metritis	3	8	0–20	0.10	0.25	−0.15
Mastitis	8	6	0–30	0.28	0.19	0.08
Lameness	2	4	0–30	0.07	0.13	−0.06
Ketosis	7	6	0–30	0.24	0.19	0.05
DA	1	1	0–30	0.03	0.03	0.00
Pneumonia	6	4	0–30	0.21	0.13	0.08
Milk fever	2	1	0–30	0.07	0.03	0.04
Anestrus	6	5	60–120	0.21	0.16	0.05
Culling rate^3^	1	0	0–60	0.04	0.00	0.04

1 Cows were randomly assigned to be treated with pegbovigrastim (PEG; 15 mg, s.c. administration 7 d before and 1 h after parturition) or control (CON; no treatment). DA = displaced abomasum.

2 Descriptive statistics were performed to determine the incidence of early-lactation diseases in the study population (n = 60; CON n = 29, PEG n = 31). Dif: proportion difference between CON group and PEG group.

3 Assessed during early lactation, between 0 and 60 DIM (n = 1: CON, n = 1; PEG, n = 0).

Pegbovigrastim has been shown to reduce mastitis incidence in dairy cows. In the present study, mastitis incidence in the PEG group was not different from the CON group during the first 30 DIM. Other studies with larger sample sizes report a significant reduction in clinical mastitis cases in animals treated with PEG (Canning et al., 2017; Ruiz et al., 2017). In those studies, PEG enhanced the microbicidal function of neutrophils from 24 h after the first administration until 7 d after the second dose (Canning et al., 2017; Van Schyndel et al., 2018). Thus, increased neutrophil populations migrating to the udder provide protection against mastitis for up to 2 wk after calving. This window of defense coincides with the first 14 d of lactation, when the risk of developing mastitis is highest.

The culling rate during the first 60 DIM in this study population was 1.67%, with 1 culling in the CON group (3.45%) due to clinical mastitis (Table 2). Previous studies, including up to 10,238 animals in their assessments, have reported that administration of PEG did not affect culling rates of Holstein dairy cows (Ruiz et al., 2017; Zinicola et al., 2018; Van Schyndel et al., 2021). In the present study, a total of 28 cows (46.67%) were culled for health or reproductive reasons in both groups during the entire course of lactation, with 38.71% in PEG and 55.18% in the CON group. The DIM at the moment of culling were at 202 ± 65 and did not differ between groups. The main causes of culling in this dairy were anestrus and subclinical mastitis (60.71% and 17.86%, respectively). Other causes included abortion, pneumonia, ketosis, and milk fever. The PEG group showed fewer animals culled due to anestrus and mastitis (50 and 17%, respectively) compared with the CON group (68 and 19%, respectively; Table 2). Nevertheless, the sample size in this study limits our ability to associate PEG with culling rates in embryo-recipient Jersey dairy cows.

**Table 2:** Culling rates and causes in early lactation (0–60 DIM) versus during the entire course of lactation in in vitro-fertilized pregnant cows induced to calve at 280 d of pregnancy^1^

Cause of culling	Group				DIM at culling		Proportion^2^					
	CON		PEG				CON		PEG		Dif	
	0–60 DIM^3^	>60 DIM^4^	0–60 DIM^3^	>60 DIM^4^	0–60 DIM	>60 DIM	0–60 DIM	>60 DIM	0–60 DIM	>60 DIM	0–60 DIM	>60 DIM
Anestrus	0	11	0	6	N/C^5^	205 ± 65	0	0.68	0	0.5	0	0.18
Mastitis	1	3	0	2	59 ± 0	197 ± 69	0.04	0.19	0	0.17	0.04	0.02
Abortion	0	0	0	3	N/C	235 ± 20^6^	0	0	0	0.25	0	−0.25
Pneumonia	0	0	0	1	N/C	122 ± 0	0	0	0	0.08	0	−0.08
Ketosis	0	1	0	0	N/C	69 ± 0	0	0.06	0	0	0	0.06
Milk fever	0	0	0	1	N/C	327 ± 0	0	0	0	0.08	0	−0.08

1 Cows were randomly assigned to be treated with pegbovigrastim (PEG; 15 mg, s.c. administration 7 d before and 1 h after parturition) or control (CON; no treatment).

2 Descriptive statistics were calculated to determine the incidence of causes of culling in the study population (n = 60: CON, n = 29; PEG, n = 31). Dif: proportion difference between CON group and PEG group.

3 Culled animals during the early-lactation assessment between 0 and 60 DIM (n = 1: CON, n = 1; PEG, n = 0).

4 Culled animals during the entire course of lactation assessment (n = 28: CON, n = 15; PEG, n = 13).

5 N/C = no culled animals during the assessed period.

6 Late-term abortion (>180 d of pregnancy).

Reproductive performance was analyzed on data from 48 cows (CON n = 21; PEG n = 27). This attrition was related to the culling of 12 animals: 11 sold in both groups due productive decisions within the farm (overpopulation) in the first week of lactation, and 1 culled due to mastitis within the first 60 DIM. The number of inseminations (times bred), days to first heat, and days to first service were similar in both groups. Calving-to-conception interval in PEG-treated cows was not significantly different from CON group; in terms of pregnancy rate, neither group was affected by treatment (PEG 87.10 vs. CON 76.32%; 95% CI 0.37, 9.49; *P* = 0.50). No differences were observed between CON and PEG-treated cows in early-lactation reproductive performance outcomes (Table 3). Previous randomized studies reported that PEG did not affect reproductive performance when days to first service and pregnancy rate were assessed in dairy cows (Zinicola et al., 2018; Van Schyndel et al., 2021; Barca et al., 2022). To our knowledge, no previous studies have evaluated the effects of PEG on days to first heat or number of inseminations. Major findings from the present study suggest that no treatment effect was observed on postpartum diseases or causes of culling, or in early-lactation reproductive outcomes.

**Table 3:** Reproductive performance in 48 in vitro-fertilized embryo-recipient Jersey cows induced to calve at 280 d of pregnancy^1^

Parameter	Group (n = 48)^2^					
	Median^3^		Dif^4^	*P*-value^5^	95% CI	
	CON (n = 21)	PEG (n = 27)			Lower^6^	Upper
Times bred	1	2	0.00	0.35	−Inf	7.05
	2.10 ± 1.58	2.15 ± 1.35				
Days to first heat	59	57	1.01	0.67	−Inf	8.99
	56.76 ± 17.52	54.89 ± 17.01				
Days to first service	59	59	4.52 × 10^5^	0.53	−Inf	3.99
	62.71 ± 12.76	63.44 ± 13.97				
Calving-to-conception interval (d)	79	85	−1.01	0.43	−Inf	18.01
	100.90 ± 53.92	101.07 ± 47.69				

1 Cows were randomly assigned to pegbovigrastim (PEG; 15 mg, s.c. administration 7 d before and 1 h after parturition) or control (CON; no treatment).

2 A total of 12 animals were culled from the initial sample of 60 cows (CON, n = 29; PEG, n = 31) due to productive decisions in the first week of lactation (n = 11) and mastitis within the first 60 DIM (n = 1).

3 Integers are the median, and values below each median are means ± SD.

4 Median of the difference between CON group and PEG group.

5 *P*-value, one-tailed (α = 0.05) Wilcoxon rank sum test at 95% confidence interval.

6 Inf = infinity.

This study has limitations. First, it was performed on a single dairy and included only Jersey cows; therefore, comparisons to previous studies require caution, as the majority of clinical trials using PEG enrolled Holstein cows. Although the sample size of this study is limited, findings are from a study population of mature, embryo-recipient Jersey cows, which are often not represented or underrepresented in larger clinical trials and observational studies evaluating the effects of PEG. Because metritis is one of the most common postpartum diseases in dairy cows and causes significant economic losses, future studies with larger sample sizes are necessary to evaluate whether PEG has negative effects on reproductive health and efficiency in embryo-recipient dairy cows.
